# WW domain-binding protein 2: an adaptor protein closely linked to the development of breast cancer

**DOI:** 10.1186/s12943-017-0693-9

**Published:** 2017-07-19

**Authors:** Shuai Chen, Han Wang, Yu-Fan Huang, Ming-Li Li, Jiang-Hong Cheng, Peng Hu, Chuan-Hui Lu, Ya Zhang, Na Liu, Chi-Meng Tzeng, Zhi-Ming Zhang

**Affiliations:** 1 Department of Breast Surgery, The First Affiliated Hospital of Xiamen University, Xiamen, Fujian 361005 People’s Republic of China; 2 Translational Medicine Research Center (TMRC), School of Pharmaceutical Science, Xiamen University, Xiamen, Fujian 361005 People’s Republic of China; 3 Key Laboratory for Cancer T-Cell Therapeutics and Clinical Translation (CTCTCT), Xiamen, Fujian 361005 People’s Republic of China; 4 INNOVA Cell Theranostics/Clinics and TRANSLA Health Group, Yangzhou, Jiangsu People’s Republic of China; 5 Teaching Hospital of Fujian Medical University, Fuzhou, Fujian 350004 People’s Republic of China

**Keywords:** WW domain, WBP2, Breast cancer, Estrogen receptor, Signaling pathway, Tyrosine kinase

## Abstract

The WW domain is composed of 38 to 40 semi-conserved amino acids shared with structural, regulatory, and signaling proteins. WW domain-binding protein 2 (WBP2), as a binding partner of WW domain protein, interacts with several WW-domain-containing proteins, such as Yes kinase-associated protein (Yap), paired box gene 8 (Pax8), WW-domain-containing transcription regulator protein 1 (TAZ), and WW-domain-containing oxidoreductase (WWOX) through its PPxY motifs within C-terminal region, and further triggers the downstream signaling pathway in vitro and in vivo. Studies have confirmed that phosphorylated form of WBP2 can move into nuclei and activate the transcription of estrogen receptor (ER) and progesterone receptor (PR), whose expression were the indicators of breast cancer development, indicating that WBP2 may participate in the progression of breast cancer. Both overexpression of WBP2 and activation of tyrosine phosphorylation upregulate the signal cascades in the cross-regulation of the Wnt and ER signaling pathways in breast cancer. Following the binding of WBP2 to the WW domain region of TAZ which can accelerate migration, invasion and is required for the transformed phenotypes of breast cancer cells, the transformation of epithelial to mesenchymal of MCF10A is activated, suggesting that WBP2 is a key player in regulating cell migration. When WBP2 binds with WWOX, a tumor suppressor, ER transactivation and tumor growth can be suppressed. Thus, WBP2 may serve as a molecular on/off switch that controls the crosstalk between E2, WWOX, Wnt, TAZ, and other oncogenic signaling pathways. This review interprets the relationship between WBP2 and breast cancer, and provides comprehensive views about the function of WBP2 in the regulation of the pathogenesis of breast cancer and endocrine therapy in breast cancer treatment.

## Background

Breast cancer is the most common of cancer among females worldwide [1]. For the treatment of early and advanced-stage breast cancer, surgery combined with radiation and chemotherapy treatment is the primary therapeutic strategy. Due to multidrug resistance, most patients exhibit poor outcome and prognosis to some chemotherapy drugs [2]. Multiple signaling pathways have been reported to be involved in the development and progression of breast cancer, such as Jak/Stat3 [3], PI3K/Akt [4], Wnt/β-catenin [5], and Hippo pathway [6]. However, there is still lack of effective biomarker to overcome drug-resistance and poor prognosis for breast cancer patients.

The WW domain is a protein-interaction domain containing two conserved tryptophan residues [7]. It is present in a large number of signaling and regulatory proteins [8, 9]. This domain has been reported for over two decades as a motif of 38 semi-conserved residues, found in a sequence of unrelated signaling and structural proteins, including dystrophin, Yes-associated protein (YAP), and two transcriptional regulators, Rsp-5 and FE65 [9–11]. The number of identified WW domains in the human proteome alone has now reached almost 100, with an additional 20 putative domains [12]. Apart from binding to proline-rich proteins, some WW domains physically interact with phosphoserine- and phosphothreonine-containing motifs [7].

The WW domain is well known as a mediator of regulatory protein complexes in various signaling networks [13]. Through the screening of synthesized WW domain libraries, WW domain has been identified as being involved in modulating the size of developing organs [14] in the Hippo signaling pathway Salvador–Warts–Hippo (or simply Hippo) by the identification of protein folding events [15–17]. Thus, this proline-rich peptide determines the biological and structural function and size of organs or in protein folding studies [18–20]. In this review, we focus on the correlation between WW domain binding protein 2 (WBP2) and breast cancer to reveal the essential functional regulatory role of WBP2 on the process of breast cancer.

### Structural motif of WBP2 and its associated binding protein

**Fig. 1:**
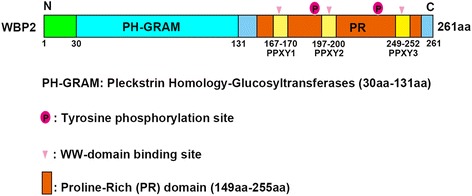
Structure of WW-domain binding protein 2. The N-terminal domain is PH-GRAM, which serves as the Rab-like GTPase activators. There are three PPxY motif within the proline-rich domain of the C-terminal region and they can recognize WW-domain containing proteins and perform a variety of biological functions. Tyrosine kinase activates Tyr192 and Tyr231 sites of WBP2 and activation of WBP2 enter the nucleus to function ability of transcriptional coactivator

WW domain-binding protein 2 (WBP2), encoded by the *WBP2* gene, was first isolated from a mouse embryo library with 32p–labeled GST-WW-YAP fusion protein and the PPxY motifs of WBP2. The molecular weight of WBP2 protein is 26–28 kD. WBP2 contains the Pleckstrin homology-glucosyltransferases (GRAM) domain, located in the N-terminal, and the proline-rich (PR) domain in the C-terminal. The PR domain of WBP2 contains three PPxY motifs, designated PPxY1–PPxY3, which have been identified as being involved in protein–protein interactions through binding with WW-domain-containing molecules [7]. WBP2 can be phosphorylated at Tyr192 and Tyr231, indicating its activation by several tyrosine kinases, and may function in certain biological effects (Fig. 1).

WBP2 can interact with several WW-domain-containing signaling molecules. With PPxY motif, WBP2 acts as a coactivator, physically binding with estrogen receptor [21]. Paired box gene 8 (Pax8), as a member of the murine Pax family of genes, has been demonstrated as required both for morphogenesis of the thyroid gland [22] and for maintenance of the thyroid differentiated phenotype that is functional differentiation of thyroid cells, measured as the expression of the differentiation markers, including thyroglobulin (Tg), thyroperoxidase (TPO), and sodium/iodide symporter (NIS) genes [23]. By immunological screening of an expression library, previous researchers have identified that WBP2 behaves as an adaptor molecule of Pax8 [24]. Furthermore, WBP2 has been isolated independently in a biochemical screen for substrates of E3 ubiquitin-protein ligase NEDD4 and Rsp5p, which are all WW domain-containing proteins, and Rsp5p protein may ubiquitinate human WBP2 in vitro [25, 26]. The PR domain of WBP2 ranks among a wide diversity of YAP ligand binding sites and the YAP–WBP2 interaction has also been reported to participate in the Hippo tumor suppressor pathway [14, 27]. PPxY motifs of WBP2 are required for the oncogenic property of WW-domain-containing transcription regulator protein 1 (TAZ), a binding partner of WBP2 mediated by WW domain [28].

Additionally, WBP2 also binds to WW1 domain, but not the WW2 domain of WW domain-containing oxidoreductase (WWOX). WWOX has been previously reported as a tumor suppressor in a diverse array of cellular activities, including growth, proliferation, apoptosis, and tumor suppression [29–32], leading to dysregulation of multiple oncogenes and tumor suppressors. Therefore, WBP2 may regulate the occurrence and progress of oncogenesis and the progress of disease through binding to the WW1 domain of WWOX gene. The WW1 domain of YAP2 also recognizes the PPxY motifs within WBP2 [33]. These binding partners are involved in multiple intracellular signaling pathways and participate in the development of a variety of diseases (Table 1), suggesting WBP2 is a key regulator in the process of several diseases.

**Table 1:** Relevant disease and signal pathway of binding partners of WBP2

Gene	Disease	Signal Pathway
Yes associated protein	Liver cancer [100], prostate carcinoma [101], lung cancer [102], breast cancer [76].	Hippo pathway [103]
Pax8	Morphogenesis of the thyroid gland [20].	Interaction with WBP2
Estrogen receptor	Breast cancer [48]	Wnt pathway [48]
Progesterone receptor	Breast cancer [17]	Interaction with YAP [17]
Rsp5p	Cancer-associated papillomavirus [21]	Ubiquitination pathway [21]
Nedd4	Liddle syndrome [104]	Ubiquitination pathway [83]
TAZ/WWTR1	Breast cancer [24]	Hippo and Wnt pathway [76]
WWOX	Breast cancer [105]	Endocrine pathway [105]

### WBP2, a coactivator of estrogen and progesterone receptor

Coactivators and corepressors play a vital role in the regulation of steroid receptor transactivation functions [34, 35]. Coactivators can act as transcriptional adaptors that mediate formation of transcriptional complexes, or modify chromatin through histone acetyl transferases (HATs) and nucleosome remodeling machineries [36]. Nuclear hormone receptor coactivators are molecules that are able to combine with enabled receptors and activate receptor-mediated transcription of target genes [34, 37–39]. Nuclear hormone receptor coactivators include the following members of the p160 family of coactivators: SRC-1, SRC-2[TIF-2/GRIP-1/NCoA-2], SRC-3[pCIP/p300-CBP/ACTR/RAC-3/TRAM-1], and E6-AP [40–45].

Ubiquitin-protein ligase E3A (E6-AP) has been reported as a novel dual-function steroid hormone receptor coactivator. Previous studies have suggested that WBP2 physically interacts with E6-AP and specifically regulates the hormone-dependent transcriptional activities of estrogen receptor (ER) and progesterone receptor (PR) [21]. WBP2 can bind to both E6-AP and ER, and all three proteins are present in a complex, both in vitro and in vivo [21]. WBP2 contains three PPxY motifs that are essential to its ability to bind with proteins containing WW domain [46]. However, only the carboxyl-terminal PPxY motif of WBP2 is required to coactivate ER. Knockdown of endogenous WBP2 results in reduced transcriptional activity of ER and PR [21]. The competition that exists between receptors limits the pool of common factors [46, 47], and coexpression of ER and WBP2 in the presence of estrogen weakens the transcriptional effect mediated by progesterone receptor. However, addition of WBP2 restores the decrease in a dose-dependent manner [21]. Studies have confirmed that WBP2 is recruited to the pS2 promoter, a well-studied estrogen-regulated gene, in a hormone-dependent manner in MCF7 cells, and these are known ER coactivators [36]. WBP2 is also essential for the accumulation of histone acetyl transferase p300, a vital histone-modifying enzyme [36]. Therefore, WBP2 strengthens ER function partly by recruiting histone modifier genes to modify the chromosome structure and enhance ER transcription. As well as E6-AP, YAP, another WW domain-containing protein, is also a transcriptional coactivator [48]. Its expression does not impact PR-mediated transactivation either in the absence or presence of hormone in HeLa cells. However, when YAP and WBP2 are coexpressed in vitro, PR-mediated transcriptional activity is dramatically increased, suggesting that YAP modulates PR transcriptional activity mainly through the existence of WBP2 protein [21].

Using complementary MALDI- and ESI-based mass spectrometry, WBP2 has been determined to be a tyrosine kinase substrate during the development of breast cancer. Additionally, RNA level of WBP2 is decreased in the MCF10AT model, which mimics the different stages of progression of breast cancer in a series of isogenic, xenograft-derived cell lines [49, 50]. However, its tyrosine phosphorylation has a higher level in breast cancer cells, in comparison with normal breast mammary epithelial cells [51]. Previously published work refers to WBP2 as a tyrosine kinase substrate, which could also traffic into the nuclei and activate ER and PR transcription [52]. Therefore, phosphorylated WBP2 is a putative coactivator for ER and can bind to other steroid hormone receptors to trigger downstream signaling transduction.

### WBP2 in breast cancer

Breast cancer is the most frequent cause of cancer death in women globally. As far as we know, ER is closely related to the development and progression of breast cancer and ERs are overexpressed in approximately 70% of breast cancer cases [53, 54]. In ER-positive cells, binding of estrogen to the ER stimulates proliferation of mammary cells, with a resulting increase in cell division and DNA replication, leading to mutations and tumor formation [55]. As a coactivator of ERα/PR, WBP2 interacts with ERα directly and activates the expression of proliferation-related target genes to function in the development and progression of breast cancer [52]. Besides ER, a number of other nuclear hormone receptors and transcriptional factors in the cells contain WW domain and WBP2 possibly interacts with these molecules to activate cancer-promoting genes in breast cancer [33].

In addition to these, WBP2 also acts as the tyrosine kinase substrate in breast tumor. The enzyme tyrosine kinase can transfer a phosphate group from ATP to protein tyrosine residues [56]. It acts as a switch module in many cellular functions, including cell growth, cell proliferation, differentiation, and apoptosis [57]. Studies suggest that 50% of oncogenes and oncogene products are tyrosine kinases. Its abnormal expression always leads to disturbances of cell proliferation regulation and induces tumorigenesis [58]. WBP2 was firstly identified to be a novel tyrosine kinase substrate in the MCF10AT model of breast cancer, and further proved to be authentic target of Epidermal Growth Factor (EGF) signaling and Iressa [51]. Iressa (gefitinib) interrupts signaling through the epidermal growth factor receptor (EGFR) in target cells and has been used in the treatment of certain breast, lung, and other cancers. Previous work has shown that EGF stimulates the expression of the tyrosine phosphorylation of WBP2 at Tyr192 and Tyr231 in WBP2-transfected HEK293T cells [51, 52]. Treatment with Iressa eliminates the increase of phosphorylated WBP2 induced by EGF in breast cancer cells, indicating that phosphorylation of WBP2 is an indispensable component of EGF signaling and the gefitinib pharmacogenomic pathway in breast cancer.

Studies show that WBP2 N-terminal-like (*WBP2NL*) gene is a testis-specific signaling protein, known as post-acrosomal sheath WW domain-binding protein [59, 60]. The N-terminal section of WBP2NL possesses a similar homology sequence to WBP2, but its C-terminal part has a PPxY motif which can bind to group I WW domain proteins [61–63]. Previous study reveals that *WBP2NL* expression is enhanced in actively dividing cancerous cell lines and this may be related to cell proliferation and tumorigenesis [64]. Utilizing RT-PCR, including semi-nested RT-PCR, researcher has determined that the overexpression of WBP2NL observed in 90% of breast cancer tissues and in MDA-MB-231 cell line, respectively [64]. These findings suggest the presence of *WBP2NL* may be a novel prognostic factor for early diagnosis of breast cancer. Moreover, the expression of several WBP2NL-related genes such as WW domain-containing E3 ubiquitin protein ligase 1 (WWP1), neural precursor cell expressed developmentally downregulated 4 (NEDD4), BCL2-associated athanogene 3 (BAG3), and WWOX is tested in both malignant breast and normal breast cancer tissues, and all are involved in the tumorigenic signal networks. Upregulation and downregulation of these genes in malignant breast cancer and normal breast cancer tissues leads us to speculate that WBP2NL potentially acts as an anti-apoptotic factor or coactivator in the development and progression of breast cancer [65]. Thus, both WBP2 and WBP2NL genes are highly related to the angiogenesis of breast carcinoma through the modulation of EGF, ER, and other downstream signaling proteins.

### Regulatory mechanism of WBP2 in breast cancer

**Fig. 2:**
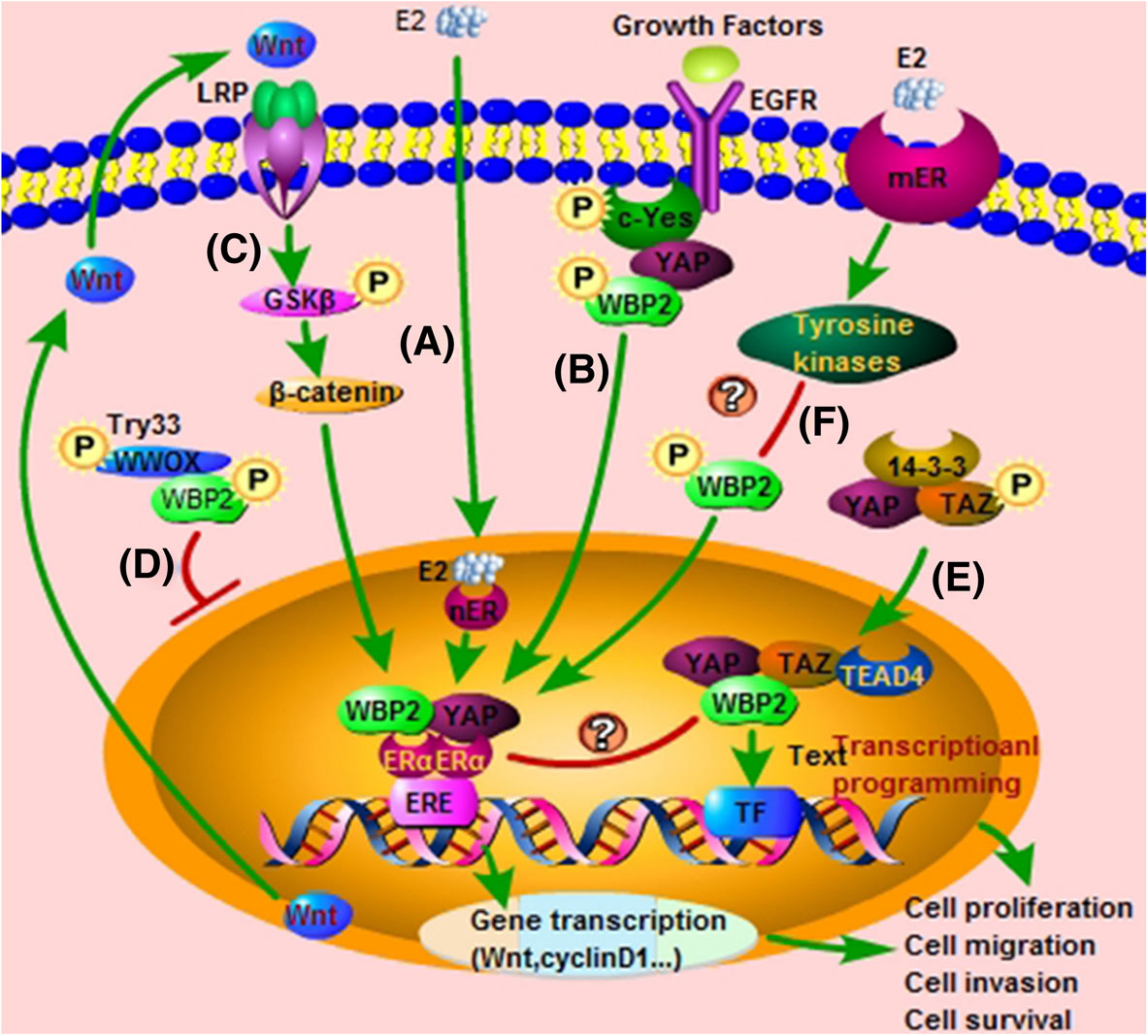
Regulatory mechanisms of WBP2 in breast cancer. **a** Once nER activated by estradiol, WBP2 then binds to YAP for stimulating the transcriptional activation of ERα. **b** WBP2 participates in the EGF signaling pathway thru binding to the complex of c-Yes and YAP in breast cancer. **c** ERα-medicated Wnt expression can activate GSKβ/β-catenin to promote the ERα-launched transcriptional programming. **d** WWOX binds PPxY domain of WBP2 and sequesters it in the cytoplasm, suppressing WBP2-mediated transcriptional functions. **e** TAZ interacts with WBP2 to improve the cell proliferation, migration, and survival through Hippo pathway in breast cancer, and the binding of them may also trigger the transcription activation of ERα. **f** Tyrosine phosphorylation of WBP2 possibly can be activated by several tyrosine kinases via the binding of E2 to mER and be drived into nuclei to function as a coactivator of ER

Endocrine therapy for breast cancer uses selective ER modulators (SERMS), such as tamoxifen, an ER antagonist in breast cancer, or aromatase inhibitors such as anastrozole [66]. ER level is used to estimate the sensitivity of breast cancer lesions to tamoxifen and aromatase inhibitors. Raloxifene, another selective ER modulator, is used as a preventive chemotherapy in women with a high risk of developing breast cancer [67]. The chemotherapeutic anti-estrogen, Faslodex, is said to be a complete antagonist of ER, enhancing its degradation and offering a new approach to the treatment of breast cancer. As a therapeutic target for breast cancer, once stimulated by estrogen, ER is capable of entering the nucleus and binding to DNA to modulate the activity of downstream genes involved in cell growth and proliferation [68]. ER is a DNA-binding transcription factor. As indicated previously, WBP2 is a coactivator of ER. Tyrosine phosphorylation of WBP2 contributes to activation of the ERα pathway and leads to an overall increase in activity of the target gene in an E2-dependent gene, such as Wnt, cyclinD1, pS2, or other target genes (Fig. 2). For instance, WBP2 is required for binding of pS2 promoter and other ER-mediated target genes to the phosphorylated form of RNA polymerase II which catalyzes the transcription of DNA to synthesize precursors of mRNA and most snRNA and microRNA [36, 69, 70].

Besides the ER signaling pathway, the Wnt signaling pathway is also strongly implicated in breast cancer [71, 72], and is likely to be an established element in WBP2-mediated breast cancer biology. The activated ERα pathway mediates the increase in Wnt expression stimulating ER transcription, which in turn modulates the activation of GSKβ and β-catenin, crucial components of the Wnt signaling pathway. Moreover, β-catenin can enter into the nuclei and further enhance the transcriptional activity of ER. Overexpression of WBP2 and WBP2 phosphor-mimic mutant is referred with Wnt reporter activities (Fig. 2a). However, FH535, a Wnt/beta-catenin inhibitor, blocks phospho-WBP2-induced tumorigenesis more dramatically than tamoxifen, in part by decreasing ERα level, suggesting tyrosine phosphorylation of WBP2 regulates ERα function in breast cancer partly via the Wnt pathway [52].

Another transcriptional coactivator, TAZ/WWTR1, encoded by the *WWTR1* gene, controls organ size and tumor suppression by restricting proliferation and promoting apoptosis [73–75]. It acts as a downstream regulatory target in the Hippo pathway. TAZ is overexpressed in primary breast cancer and interacts with WBP2 via the WW-domain of TAZ and the C-terminal PPxY motif of WBP2, to enhance the migration of breast cancer cell lines [28, 76–78]. TAZ is significant for its ability to transform MCF10A and NIH3T3 cells into mesenchymal like cells through epithelial mesenchymal transformation process (EMT). Limiting the expression of endogenous WBP2 suppresses, whereas overexpression of WBP2 enhances, the transformation and transcription-promoting ability of TAZ, suggesting WBP2 is a crucial part of TAZ-launched tumor cell metastasis. YAP is a putative ER coactivator and its coactivation function relies on the coexpression of functional WBP2 [21], Moreover, WBP2 is a YAP-interacting factor and can increase the transforming capacity of YAP in both MCF10A and NIH3T3 cells [7, 28]. Interestingly, phosphorylated WBP2 can promote transcriptional programs, including cell growth, migration, and apoptosis, by combining with TAZ and YAP complexes in the nucleus (Fig. 2e). Emerging studies also show that Wnt signaling promotes breast cancer by blocking ITCH-mediated (an ubiquitin-conjugating enzyme) ubiquitin-dependent degradation of the YAP/TAZ transcriptional coactivator WBP2, suggesting WBP2/ITCH signaling functions to bridge the complex Wnt and Hippo signaling networks in breast cancer [79].

WWOX has recently been found to be highly expressed in mammary gland, prostate, and ovary. As a typical tumor suppressor, the status of WWOX expression is strongly coupled with breast cancer progression and prognosis. In addition, studies show that phosphorylated WWOX is able to physically bind to the PPxY motif of WBP2 and inhibit the ER transactivation pathway, further attenuating the process of breast cancer [21, 80] (Fig. 2d). These studies suggest that WBP2, via the ER, Wnt, Hippo, and WWOX pathways, plays a critical role in breast cancer development. A considerable amount of work remains to further illuminate its function in the progression of breast cancer.

## Discussion

WBP2 has been identified as a binding partner of several proteins containing WW domain including YAP [7, 81], TAZ [82, 83], WWOX1 [30, 84, 85], Nedd4 [86], Pax8 [24], and E6-AP [21]. The WW domain in these proteins forms a binding pocket for the PPxY-motif of WBP2, and the interaction between WBP2 and WW-domain-containing proteins activates downstream signaling such as the Hippo and ER pathways. Studies also show that WBP2 may be a crucial downstream element of the Hippo pathway [14]. Owing to its proline-rich domain, WBP2 may combine with other WW-domain-containing proteins and is involved in regulating diverse cellular functions. For instance, protein salvador homolog 1, encoded by the *SAV1* gene, contains 2 WW domains and a coiled-coil region [87]. Experiments show that it can bind to mammalian sterile 20-like kinase 1 (MST1) or hematopoietic cell-specific protein 1 (HS1)-associated protein X-1 (HAX1) and promotes MST1-induced apoptosis or reduces the anti-apoptotic effects of HAX1 [88]. This gene potentially acts as a tumor suppressor in humans and mice. It is possible that WBP2 recognizes the WW domain of protein salvador homolog 1 and functions as a crucial component in SAV1-induced apoptosis. Other WW-domain-containing proteins may interact with WBP2 to function in human breast disease or other diseases.

Current studies on WBP2 mostly concentrate on its function as an ER and PR transactivation coactivator. The coactivation function of WBP2 is modulated by tyrosine phosphorylation, including Tyr192 and Tyr231. Other WBP2 regions may be equally important for its coactivator ability [46, 86]. Estradiol is not only a nuclear ER agonist, but also acts as an agonist of membrane ER, via which it can launch a variety of rapid, non-genomic effects [89]. These rapid estrogen-mediated cellular responses to physiological concentrations of estrogens are transmitted via enzymatic pathways and ion channels through the activation of what are generically denoted as membrane-associated ERs (mERs). Generally, mERs are complex-containing signal proteins, such as insulin-like growth factor (IGF) and EGF receptor, Ras protein, adaptor protein Shc, and non-receptor tyrosine kinase c-Src. mER plays a central role in this complex [90]. Once E2 is bound to mERs, these signalosomes, which participate in activation of the tyrosine kinase pathway, for instance, c-Src, may stimulate the tyrosine phosphorylation of WBP2, a novel substrate of tyrosine kinase. Activated WBP2 enters into the nuclei to function as a coactivator of ER and promote transcriptional programming (Fig. 2f).

As a transactivation coactivator of ER, WBP2 plays an effective role in ER-positive tissue or cell lines, such as breast tissue or ER-positive breast cancer cell lines. Additionally, researchers have identified WBP2 as a novel mediator in the putative E2-EGFR-WBP2-Wnt-ERα pathway [52]. However, our preliminary data show that WBP2 is also overexpressed in ER-negative breast cancer cell lines, such as MDA-MB-231(unpublished data). Probably the most significant finding from research is that WBP2 is not only dependent on the existence of ER/ERα, but can also activate other transcription factors, including E2F-1 [91], NF-KB [92], and STAT6 [93], independent of ER. These are all nuclear receptor family members and are involved in multiple biological functions, indicating that WBP2 may participate in several essential signal transductions. Recent research has shown that WBP2 is also required for normal glutamatergic synapses in the cochlea in an ER-dependent manner, suggesting that WBP2 may participate in the molecular pathway linking hearing impairment to hormonal signaling, this knowledge provides new therapeutic targets for treatment of hearing impairment [94].

The focus of endocrine therapy for breast cancer is at the ER level. Tamoxifen is a selective estrogen receptor modulator [95]. It is currently used for the treatment of both early and advanced ER-positive breast cancer in pre- and post-menopausal women [96]. However, increased Her2 or other causes can promote the proliferation of cancer cells against endocrine therapy for breast cancer and induce tamoxifen resistance. WBP2 can trigger ER transcription and further promote cell growth; such associations may be powerful enough to label WBP2 a novel key modulator of tamoxifen resistance [97–99]. Conversely, the interaction between WWOX and WBP2 inhibits the ER transactivation pathway [21, 80], indicating that WBP2 may be a generalist in regulating ER signal transduction and tamoxifen resistance (Fig. 2). Although tamoxifen is an ER antagonist in breast tissue, it acts as an agonist on the endometrium and studies have confirmed that it can induce endometrial cancer in some women [100]. Thus, the use of tamoxifen has a high risk of adverse side effects. WBP2 may participate in the development of some uterine and ovarian cancer diseases that have a strong relationship with ER.

WBP-2 localizes on the chromosome 17q25 region, which is known as being involved in certain forms of human carcinogenesis, such as chronic myelogenous leukemia (CML). WBP2 potentially acts as a carcinogen in several organs in vivo [27]. Fumonisin B_1_ (FB_1_) is a mycotoxin produced by the phytopathogenic fungus *Fusarium moniliforme*, which structurally resembles sphingoid bases [101]. It contributes to primary hepatocellular carcinoma in rats and may also play a vital carcinogenic role in several human cancers [102]. In addition, in vivo study suggests FB_1_ also induces cell apoptosis in rat kidney [103]. Utilizing a PCR-based subtraction approach, Zhang identified 8 genes, including WBP2, that mediate the lethal effects that FB_1_ has on monkey kidney CV-1 cells [101]. The GRAM domain within the N-terminal of WBP2 serve as Rab-like GTPase activators, and our experiments focus on the crucial role of WBP2 in the development of glioma. The data showed that WBP2 promotes proliferation and metastasis of glioma cells by affecting the Embden–Meyerhof pathway (unpublished data). This indicates that WBP2 is not only a hub protein in breast cancer, but also a key regulator in other malignant carcinomas.

## Conclusion

Most of the research on functions of WBP2 undertaken to date has distinctly clarified its role in breast cancer. Significant advances have been made in the understanding of the biological, structural, and chemical nature of WBP2, but as a scaffolding protein, its other biological roles in organs or diseases needs more research. Exploration of the other functions of WBP2 may provide more clues to the vital role of WBP2 in the development and progress of breast cancer.

## Abbreviations

BAG3: BCL2-associated athanogene 3
CML: Chronic myelogenous leukemia
E6-AP: Ubiquitin-protein ligase E3A
EGF: Epidermal growth factor
EGFR: Epidermal growth factor receptor
ER: Estrogen receptor
GRAM: Glucosyltransferases
HAX1: Hematopoietic cell-specific protein 1 (HS1)-associated protein X-1
IGF: Insulin-like growth factor
MDA-MB-231 cell: Triple-negative breast cancer
mERs: Membrane-associated ERs
MST1: Mammalian sterile 20-like kinase 1
NEDD4: An E3 ubiquitin-protein ligase
Pax8: Paired box gene 8
PR: Progesterone receptor
TAZ: WW-domain-containing transcription regulator protein 1
WBP2: WW domain-binding protein 2
WBP2NL: WBP2 N-terminal-like gene
WWOX: WW-domain-containing oxidoreductase
WWP1: WW domain-containing E3 ubiquitin protein ligase 1
Yap: Yes kinase-associated protein
